# Association between length of hospital stay and implementation of discharge planning in acute psychiatric inpatients in Japan

**DOI:** 10.1186/s13033-015-0015-9

**Published:** 2015-05-30

**Authors:** Miharu Nakanishi, Junko Niimura, Michika Tanoue, Motoe Yamamura, Toyoaki Hirata, Nozomu Asukai

**Affiliations:** Mental Health and Nursing Research Team, Tokyo Metropolitan Institute of Medical Science, Setagaya-ku, Tokyo, Japan; Mental Health and Psychiatric Nursing, Graduate School of Health Care Sciences, Tokyo Medical and Dental University, Bunkyo-ku, Tokyo, Japan; Division of Nursing Sciences, Graduate School of Human Health Sciences, Tokyo Metropolitan University, Arakawa-ku, Tokyo, Japan; Chiba Psychiatric Medical Centre, Chiba-shi, Chiba Japan; Research Project for Mental Health Promotion, Tokyo Metropolitan Institute of Medical Science, Setagaya-ku, Tokyo, Japan

## Abstract

**Background:**

Japan has introduced an acute psychiatric care unit to the public healthcare insurance program, but its requirement of a shorter length of stay could lead to discharges without proper discharge planning. The aim of this study was to examine the association between the implementation of discharge planning and the length of stay of acute psychiatric inpatients in Japan.

**Methods:**

This retrospective cross-sectional study included 449 patients discharged from the ‘psychiatric emergency ward’ of 66 hospitals during a two-week period from March 7 to 20, 2011. The assigned nurse or nursing assistant for each patient provided information on the implementation of discharge planning in the hospital stay.

**Results:**

Approximately one quarter of the 449 patients (n = 122) received no support for coordination with post-discharge community care resources. The 122 patients who had received no support for community care coordination had a significantly lower mean age at admission, a shorter length of stay, and a higher rate of either no follow-up or unidentified post-discharge outpatient service than the other 327 patients. Multilevel linear regression analysis demonstrated a significantly greater length of stay among patients who were older, those who had a primary diagnosis of schizophrenia, those who were admitted compulsorily, those who received hospital outpatient services, and those who received community care coordination support from the assigned nurse or nursing assistant. The implementation of support for community care coordination did not indicate a significant association with these factors, which have been related to an increased risk of psychiatric readmission.

**Conclusion:**

Patients to whom the assigned nurse or nursing assistant provided support on community care coordination experienced a significantly greater length of hospital stay. The implementation of support for community care coordination did not indicate a significant association with these factors, which have been related to an increased risk of psychiatric readmission. The mental health policy should increase focus on discharge planning in the acute psychiatric setting to enhance a link between psychiatric inpatient care and post-discharge community care resources.

## Background

Historically, Japan has a larger number of psychiatric hospitals than other Organisation for Economic Cooperation and Development (OECD) countries. Moreover, the length of stay in psychiatric beds in Japan appears strikingly longer than other OECD countries [1]. In 2004, the Ministry of Health, Labour, and Welfare announced a policy for the transition from psychiatric inpatient care to community-based mental health services [2]. Nevertheless, the mental health care system in Japan has predominantly remained dependent on hospital-based services, with only a slight decline in the length of stay in psychiatric hospitals during the last decade. The National Patient Survey reported an average of length of stay of 385.7 days in 2002 [3] and 341.6 days in 2011 [4].

Several patient characteristics have been identified as factors related to a longer length of stay in psychiatric inpatient care; such factors include male sex [5–8], schizophrenic and delusional disorders [6, 7, 9], physical comorbidity [8], younger age of onset of mental disorder [8], and less adherence to medication [10]. However, length of stay depends on the service system rather than individual patient characteristics [11, 12].

The national government has initiated several policies to promote the transition from psychiatric inpatient care to community-based mental health services. Under the public healthcare insurance program, the Ministry of Health, Labour, and Welfare introduced an acute psychiatric care unit as well as an additional benefit schedule to pay for discharge planning in psychiatric inpatient care [13]. Acute psychiatric care units under the public healthcare insurance program are designed to discharge inpatients within 3 months. Psychiatric acute care hospitals had an average of length of stay of 54.4–79.7 days in 2012 [14]. Discharge planning provides the critical link between inpatient treatment and community-based post-discharge care [15, 16]. The additional benefit schedule for discharge planning is aimed at improving discharge of long-stay inpatients, paying only for psychiatric long-term care units. However, discharge planning is required not only for long stays but also in acute care settings to prevent long stays and readmission in new inpatients. A shorter length of stay without proper discharge planning would increase risk for readmission [17, 18]. Follow-up outpatient services prevent readmissions of psychiatric patients after discharge [19]. Provider communication between inpatient treatment and outpatient care plays a key role in preventing early readmission [20]. However, the requirement of a shorter length of stay could lead to discharges from acute psychiatric inpatient care without proper discharge planning. Thus, the aim of the present study was to examine the association between the implementation of discharge planning and length of hospital stay of acute psychiatric inpatient care in Japan. We hypothesised that enhanced implementation of discharge planning would require a relatively greater length of stay for acute psychiatric inpatients.

## Methods

### Design

This study was conducted using a retrospective cross-sectional design.

### Setting

Eligible institutions consisted of 80 psychiatric hospitals with a ‘psychiatric emergency ward’ in the list compiled by the Japanese Association for Emergency Psychiatry at the end of August 2010. The psychiatric emergency ward is certified as an acute psychiatric care unit under the fee schedule for the public healthcare insurance program; the ward has a high staffing requirement, including one or more full-time designated psychiatrists, two or more full-time psychiatric social workers, and one or more nurses per ten patients.

Each hospital was asked to rate patients who were discharged from the hospital’s psychiatric emergency ward during a two-week period from March 7th to 20th, 2011. Each hospital was also asked to exclude patients who were discharged to another unit in the same hospital, another hospital, or a medical care facility under the public long-term care insurance program.

### Participants

A total of 561 patient-level questionnaires were collected from 66 hospitals, an 82.5% response rate. Among the 561 questionnaires, 112 questionnaires were excluded because of incomplete information. The final sample for analysis consisted of the remaining 449 questionnaires from 66 hospitals. In contrast to the 112 excluded patients, the 449 included patients had a higher rate of discharge to their own home where they lived alone [*χ*^2^ (5) = 12.55, *p* = 0.028]. The 66 hospitals included in this study had an average of 63.4 beds in emergency care units. One-third of the 66 hospitals (n = 20) were located in the Kanto region. There was no significant difference from the 14 hospitals that did not respond to the survey either in number of beds in emergency care wards [*t* (17.46) = 0.05, *p* = 0.958] or in distribution of regions [*χ*^2^ (6) = 3.94, *p* = 0.685].

### Procedures

The questionnaires were administered over a two-month period from March to April 2011. A set of paper questionnaires was mailed to each participating hospital. The completed questionnaires were also collected by mail.

An assigned nurse or nursing assistant was asked to read the instructions and to answer the questions independently. The set of questionnaires had an introductory section, including a description of the purpose of the study, an explanation of the voluntary nature of participation, and an assurance of anonymity for the patients and responding hospitals. The participating hospitals were not required to sign consent forms; returning the questionnaire implied consent. Informed consent was not sought from the patients because our observation was based on retrospective reports from nurses about the patients who had already been discharged from the hospital.

### Measures

The questionnaire collected information on patient characteristics, implementation of discharge planning, social functioning at discharge, and length of stay.

Discharge planning consisted of seven domains and 34 items (Table 1). These domains and items were reviewed by a working group that included researchers and practitioners from a psychiatric hospital. The domains were developed based on categories of the psychiatric nursing intervention classification system in Japan [21]. The items were intended to cover a variety of discharge planning [22], the concept of the therapeutic nurse-client relationship [23], and family involvement [24]. The assigned nurse or nursing assistant retrospectively rated implementation of the activity during the early-mid phase of the hospital stay and in preparation for discharge (1 = implemented, 0 = not implemented). The early-mid phase was defined as the duration from admission to reduction in acute symptoms (e.g., the patient is moved from a seclusion room to a shared bedroom). Because the present study focused on the link between inpatient treatment and community-based post-discharge care, patients were divided into the following two groups: 122 patients who had received no support for seven activities in coordination with post-discharge community care resources both during the early-mid phase and in preparation for discharge, and the remaining 327 patients who had received any community care coordination support.

**Table 1:** Discharge planning in the early-mid phase of the hospital stay and preparation for discharge

Activity	*n* (%)	
	Early-mid	Preparation
Psychotropic medication management		
Explore the patient’s view on medication	295 (65.7)	308 (68.6)
Share with the patient why medication will be continued, and how to take medication post-discharge	176 (39.2)	289 (64.4)
Education about self-administration of medication	36 (8.0)	104 (23.2)
Explain strategies to address post-discharge side effects of medication	103 (22.9)	205 (45.7)
Consult with an assigned physician on expected difficulties in post-discharge medication management	105 (23.4)	164 (36.5)
Disease management		
Explain mechanism of the disease and symptoms	119 (26.5)	169 (37.6)
Discuss identification of each symptom in accordance with the patient’s perceived experience of disease	237 (52.8)	271 (60.4)
Discuss the cause of hospitalisation	285 (63.5)	295 (65.7)
Explore the patient’s feelings and wishes in his/her life with the disease	193 (43.0)	281 (62.6)
Explain why hospital staff (in outpatient service) and the patient will meet regularly post-discharge	169 (37.6)	331 (73.7)
Verify the date of outpatient service and means of transportation	95 (21.2)	300 (66.8)
Symptoms management		
Discuss identifying triggers that increase symptoms	191 (42.5)	280 (62.4)
Discuss identifying signs of deterioration	140 (31.2)	244 (54.3)
Develop and share post-discharge strategies to address deterioration post-discharge	106 (23.6)	261 (58.1)
Ensure means of post-discharge access to healthcare agency when the symptoms worsen	82 (18.3)	237 (52.8)
Advice about coping with symptoms	172 (38.3)	264 (58.8)
Support for personal relationships		
Facilitate self-exposure based on assertion training	46 (10.2)	74 (16.5)
Advice and intervention for relationship building	129 (28.7)	173 (38.5)
Family support		
Contact with family members to provide emotional support	161 (35.9)	183 (40.8)
Complement the information that physician provided to family members	170 (37.9)	200 (44.5)
Family involvement		
Advice about communication with the patient	129 (28.7)	170 (37.9)
Debrief responses and feelings when visiting the patient or the patient visits them	199 (44.3)	266 (59.2)
Respond to their concerns about the patient’s symptoms and challenging behaviours	182 (40.5)	210 (46.8)
Inform current condition and prognosis of the patient	173 (38.5)	198 (44.1)
Share regarding the disease and medication	135 (30.1)	183 (40.8)
Refer to peer support group available in the community or hospital	46 (10.2)	59 (13.1)
Explain why medication and outpatient service will be continued, and how to address deterioration and crisis	93 (20.7)	195 (43.4)
Coordination with post-discharge community care resources		
Consult on social rehabilitation and participation to facilitate the patient’s wishes	88 (19.6)	211 (47.0)
Contact with family members to explore the patient’s post-discharge place in daily life	96 (21.4)	203 (45.2)
Trial participation in day-care, workshop, or Alcoholics Anonymous	48 (10.7)	86 (19.2)
Visit planned post-discharge residence	38 (8.5)	59 (13.1)
Help the patient collect necessities for post-discharge daily life	73 (16.3)	148 (33.0)
Multidisciplinary meeting to share information on social rehabilitation, participation, and place in daily life	97 (21.6)	153 (34.1)
Meeting with in-hospital workers and community service providers	55 (12.3)	94 (20.9)

Length of stay was defined as the number of days between admission and discharge.

Social functioning at discharge was retrospectively evaluated by the Japanese version of Global Assessment of Functioning (GAF) scale [25]. The GAF provides a summary score reflecting the level of psychological, social, and occupational functioning on a scale from 1 to 10. Satisfactory validity was reported [26].

Patient characteristics included age, sex, education level, working status before admission, primary diagnosis, juridical basis of admission, total number of psychiatric hospitalisations, presence of physical comorbidity, current place of residence, and follow-up outpatient service after discharge. Current place of residence was divided into living alone at own home, cohabitation with family, residential care facility, readmitted to hospital from home, dead shortly after discharge, or unidentified place of residence. ‘Residential care facility’ included daily life adjustment training facilities (engoryou), welfare homes (fukusi-home), group homes, and other residential facilities (nyuusyo-shisetsu). Follow-up outpatient services after discharge assessed whether the discharged patient received follow-up services. The questionnaire also included age at first presentation to mental health services. However, as many respondents did not indicate the patient’s age at first presentation (64 of 449, 14.3%), this was excluded from the analysis.

### Ethical considerations

To preserve respondent anonymity, identification numbers were assigned to hospitals. The study was approved by the Ethics Committee of the Tokyo Institute of Psychiatry.

### Statistical analysis

Differences between the 122 patients who had received no support for community care coordination and the remaining 327 patients were analysed based on patient characteristics and length of stay. Because some categories of primary diagnosis had a small number of patients (<10%), diagnoses were divided into schizophrenia, affective disorders, or other. Work status was divided into the following three categories: engaged in work, not engaged in work, and unidentified working status. The length of stay was transformed using a natural logarithm due to the highly skewed distribution.

Multilevel linear regression analysis was used using the natural logarithm of length of stay as the dependent variable and implementation of discharge planning as the independent variable. The model also included patient characteristics as independent variables.

Because data were taken from multiple patients from each hospital, multilevel modelling was used for multivariate analyses. The models included random effects for each hospital to account for within-hospital correlations. All statistical analyses were conducted using Stata SE for Windows, version 13.0 (StataCorp, College Station, Texas, USA). The two-tailed significance level was set at 0.05.

## Results

### Patient characteristics

The mean age at admission was 44.9 years. Half of respondent patients were men, not engaged in work, and had a primary diagnosis of schizophrenic disorders. The majority of patients were discharged to cohabitation with family. The average length of stay was 49.0 days (*SD* = 50.9) (Table 2).

**Table 2:** Characteristics of 449 patients discharged from emergency psychiatric care units in Japan

Patient characteristic	Mean (SD) or *n* (%)	
Age at admission, year (median = 43.0)	44.9	(17.3)
Sex, male	206	(45.9)
Education level		
Junior high school	119	(26.5)
High school	175	(39.0)
Vocational school, college	49	(10.9)
University	69	(15.4)
Unidentified	37	(8.2)
Working status before the admission		
Full-time	56	(12.5)
Part-time	47	(10.5)
Sheltered employment	12	(2.7)
Employment, unspecified	28	(6.2)
Family business	54	(12.0)
Not engaged in work	234	(52.1)
Unidentified	18	(4.0)
Primary diagnosis, ICD-10		
Schizophrenia, schizotypal, and delusional disorders (F20–F29)	203	(45.2)
Mood (affective) disorders (F30–F39)	115	(25.6)
Psychoactive substance use (F10–F19)	26	(5.8)
Organic (F00–F09)	25	(5.6)
Neurotic, stress-related and somatic (F40–49)	24	(5.4)
Other categories	56	(12.5)
Juridical basis of admission, compulsory	347	(77.3)
Total number of psychiatric admissions	3.1	(4.3)
Physical comorbidity	103	(22.9)
Length of stay, day	49.0	(50.9)
Social functioning at discharge (1–10)	6.6	(1.7)
Current place of residence		
Lives alone at own home	69	(15.4)
Cohabitation with family	306	(68.2)
Residential care facility	26	(5.8)
Readmitted to hospital from home	20	(4.5)
Dead shortly after discharge	2	(0.5)
Place of residence unidentified	26	(5.8)
Follow-up outpatient service after discharge		
Followed by outpatient service of the hospital	309	(68.8)
Followed by outpatient service of other hospital	123	(27.4)
No follow-up with post-discharge outpatient service	7	(1.6)
Unidentified	10	(2.2)

### Implementation of discharge planning

In the early-mid phase of the hospital stay, more than half of assigned nurses or nursing assistants of the 449 patients discussed with patients their view on medication (65.7%), the cause of hospitalisation (63.5%), and identification of each symptom in a manner consistent with the patient’s perceived experience of disease (52.8%). Less than one-tenth of nurses provided education about self-administration of medication to the patient (8.0%) or visited the planned post-discharge residence (8.5%; Table 1). The 449 patients had an average of 9.9 activities (standard deviation 8.0) in the early-mid phase of the hospital stay and 16.2 (9.6) at preparation for discharge. All seven domains showed a significant increase in the total number of implemented activities from the early-mid phase of the hospital stay to preparation for discharge [*t* (448), 4.19–15.19; p < 0.05]. Approximately one quarter of the 449 patients (*n* = 122) had received no support for coordination with post-discharge community care resources.

### Patient characteristics and support on community care coordination

The 122 patients who had received no support for community care coordination had a significantly lower mean age at admission, a shorter length of stay, and a higher rate of either no follow-up or unidentified post-discharge outpatient service than the other 327 patients (Table 3).

**Table 3:** Comparison between 122 patients who did not and 327 patients who did receive community care coordination support

Patient characteristic, mean (SD) or *n* (%)	Community care coordination support			
	No (*n* = 122)	Yes (*n* = 327)	Test statistic	*p* value
Age at admission, year	41.5 (17.1)	46.2 (17.3)	t (218.82) = 2.55*	0.011
Sex, male	52 (42.6)	154 (47.1)	*χ* ^2^ (1) = 0.72	0.398
Education level			*χ* ^2^ (4) = 2.96	0.564
Junior high school	27 (22.1)	92 (28.1)		
High school	123 (37.6)	52 (42.6)		
Vocational school, college	11 (9.0)	38 (11.6)		
University	20 (16.4)	49 (15.0)		
Unidentified	25 (7.7)	12 (9.8)		
Working status before the admission			*χ* ^2^ (2) = 4.58	0.101
Engaged in work	46 (37.7)	151 (46.2)		
Not engaged in work	68 (55.7)	166 (50.8)		
Unidentified	8 (6.6)	10 (3.1)		
Primary diagnosis			*χ* ^2^ (2) = 0.14	0.932
Schizophrenic	56 (45.9)	147 (45.0)		
Affective	32 (26.2)	83 (25.4)		
Other	34 (27.9)	97 (29.7)		
Compulsory admission	97 (79.5)	250 (76.5)	*χ* ^2^ (1) = 0.47	0.492
Total number of psychiatric admissions	3.0 (4.2)	3.1 (4.3)	*t* (218.90) = 0.20	0.841
Physical comorbidity	25 (20.5)	78 (23.9)	*χ* ^2^ (1) = 0.57	0.451
Length of stay, day	33.2 (46.2)	54.9 (51.4)	*t* (239.65) = 4.30*	<0.001
Social functioning at discharge (1–10)	6.4 (1.8)	6.7 (1.6)	*t* (200.86) = 1.79	0.075
Current place of residence			*χ* ^2^ (5) = 9.00	0.109
Lives alone at own home	14 (11.5)	55 (16.8)		
Cohabitation with family	84 (68.9)	222 (67.9)		
Residential care facility	6 (4.9)	20 (6.1)		
Readmitted to hospital from home	6 (4.9)	14 (4.3)		
Dead shortly after discharge	2 (1.6)	0 (0.0)		
Place of residence unidentified	10 (8.2)	16 (4.9)		
Follow-up outpatient service after discharge			*χ* ^2^ (2) = 17.61*	<0.001
Followed by outpatient service of the hospital	75 (61.5)	234 (71.6)		
Followed by outpatient service of other hospital	35 (28.7)	88 (26.9)		
No follow-up/unidentified	12 (9.8)	5 (1.5)		

* Significant at *p* < 0.05.

### Factors related to the length of stay

The multilevel linear regression analysis demonstrated a significantly greater length of stay among older patients and those with a primary diagnosis of schizophrenia, compulsory admission, hospital outpatient follow-up services, and who had received support for community care coordination. The random effect of hospital accounted for <1% of total variance (Table 4).

**Table 4:** Linear regression analysis of the natural logarithm of length of stay

	Coefficient	95% CI	*p* value
Intercept	2.41	1.89–2.93	<0.001
Fixed effect			
Age at admission, year	0.01*	0.01–0.02	<0.001
Sex, male	0.11	−0.05–0.28	0.183
Education level (junior high school = 0)			
High school	−0.02	−0.23–0.19	0.848
Vocational school, college	0.14	−0.16–0.44	0.361
University	0.04	−0.23–0.31	0.771
Unidentified	−0.16	−0.49–0.17	0.337
Working status before admission (not engaged in work = 0)			
Engaged in work	−0.15	−0.33–0.02	0.083
Unidentified	−0.23	−0.66–0.19	0.285
Primary diagnosis (other = 0)			
Schizophrenic	0.40*	0.20–0.59	<0.001
Affective disorder	0.18	−0.05–0.41	0.129
Compulsory admission	0.47*	0.27–0.67	<0.001
Total number of psychiatric admissions	−0.01	−0.03–0.01	0.267
Physical comorbidity	0.04	−0.17–0.24	0.714
Current place of residence (cohabitation with family = 0)			
Lives alone at own home	−0.18	−0.42–0.05	0.130
Residential care facility	0.01	−0.35–0.37	0.921
Readmitted to hospital from home	−0.18	−0.59–0.22	0.372
Dead shortly after discharge	−0.49	−1.72–0.74	0.435
Place of residence unidentified	−0.21	−0.56–0.15	0.258
Social functioning at discharge	−0.03	−0.08–0.02	0.255
Follow-up outpatient service after discharge (follow-up outpatient service by the hospital = 0)			
Follow-up outpatient service of other hospital	−0.35*	−0.54 to −0.17	<0.001
No follow-up/unidentified	−1.17*	−1.63 to −0.72	<0.001
Received support on community care coordination	0.61*	0.43–0.80	<0.001
Random effect			
Residual	0.73	0.63–0.84	
Intercept: hospital	0.003	10^−11^ × 0.28 − 23,291.62	
Intra-class correlation coefficient (ICC)	<0.001		
Fitness of model			
χ^2^ (23)	178.61		<0.001
Log likelihood	−566.48		
Akaike’s information criterion (AIC)	1,182.97		

* Significant at *p* < 0.05.

## Discussion

### Main findings of the study

As expected, the implementation of discharge planning demonstrated a negative association with length of stay. A significantly longer length of stay was observed among patients who had received support on coordination with post-discharge community care resources. Furthermore, a greater length of stay was observed among patients who received post-discharge follow-up by the hospital outpatient service. Patients who did not receive community care coordination support were discharged after a shorter length of stay from the hospital with either unidentified outpatient services or no follow-up. These results suggest that because of the requirement of a shorter length of stay, some patients may have been discharged from acute psychiatric hospitals without proper discharge planning. The public healthcare insurance program requires that at least 40% of annual admissions to psychiatric emergency units are new patients, although the readmission rate of discharged inpatients is not taken into account under the fee schedule. The mental health policy should increase focus on discharge planning in the acute psychiatric setting to enhance a link between psychiatric inpatient care and post-discharge community care resources.

Among the 34 discharge planning activities, only three were implemented to half of the 449 patients during the early-mid phase of the hospital stay, although discharge planning should begin at the time the inpatient is admitted to the psychiatric unit. The implementation of discharge planning significantly increased in preparation for discharge. However, coordination with post-discharge community care resources was never administered to a quarter of the 449 patients. The nurses would have determined the implementation of discharge planning according to individual patient needs. Meanwhile, the implementation of support for community care coordination did not indicate a significant association with compulsory admission or schizophrenic disorder, which have been related to an increased risk of psychiatric readmission [27]. As the psychiatric emergency ward has to discharge inpatients within 3 months, it may be that inpatient treatment had a greater focus on the management of acute psychosis. In addition, the majority of our sample patients were admitted involuntarily. In the psychiatric emergency ward, at least 60% of all admissions should be involuntary admissions. Thus, a longer stay would have required establishment of a therapeutic patient-nurse relationship.

The length of stay in acute psychiatric care was significantly associated with a primary diagnosis of schizophrenic disorder and compulsory admission. This is in agreement with previous research in Western psychiatric inpatient care settings [5–7, 9]. However, there was no significant association with male sex and physical comorbidity. These findings are in contrast with a previous study regarding patient characteristics and longer length of stay [5–8]. Furthermore, in contrast from previous findings [6, 7], it is notable that the length of stay in the psychiatric emergency ward did not differ between the hospitals. The universal benefit schedule strict defines the care setting in the psychiatric emergency ward under the public healthcare insurance program. Therefore, care settings would not differ between units. Factors relating to length of stay should be explored among inpatients in other psychiatric care settings as well as psychiatric emergency wards in Japan.

## Limitations

The present study has the following limitations. First, while the response rate was large, there may have been a responder bias. Second, the patients in our sample had a greater rate of discharge to their own residence than those excluded from the analyses. Third, the implementation of nursing care was evaluated by one assigned nurse or nursing assistant of each patient. Discharge planning is based on a multidisciplinary approach; hence, information on discharge planning should be collected with multi-professional views. Fourth, the assessment did not collect information on patient medication compliance and social functioning at admission, both of which have previously been associated with length of stay [5]. Finally, our observation was based on a retrospective and cross-sectional evaluation that provided information only about those who were discharged. Length of stay in psychiatry is suggested to follow a hazard-based distribution [28]. Future research should examine the effect of discharge planning on readmission rate of psychiatric inpatients as well as length of stay in a prospective study design.

Despite these limitations, the present study is the first to present an association between the implementation of discharge planning and length of stay for acute psychiatric care using national representative data.

## Conclusions

The present study examined the association between the implementation of discharge planning and the length of stay of acute psychiatric inpatients in Japan. Patients to whom the assigned nurse or nursing assistant provided support on community care coordination showed a significantly greater length of hospital stay. The length of stay in acute psychiatric care was also significantly associated with a primary diagnosis of schizophrenic disorder and compulsory admission. The implementation of support for community care coordination did not indicate a significant association with these factors, which have been related to an increased risk of psychiatric readmission. The mental health policy should increase focus on discharge planning in the acute psychiatric setting to enhance a link between psychiatric inpatient care and post-discharge community care resources.
